# Perception of violence by transvestigender people in Brazil and its implications for primary health care

**DOI:** 10.1590/S2237-96222024v33e2024283.especial.en

**Published:** 2025-01-10

**Authors:** Mônica Machado Cunha e Mello, Rodrigo Otávio Moretti-Pires, Marcos Claudio Signorelli

**Affiliations:** 1 Universidade Federal de Santa Catarina, Programa de Pós-Graduação em Saúde Coletiva, Florianópolis, SC, Brasil; 2 Centro Universitário para o Desenvolvimento do Alto Vale do Itajaí, Curso de Psicologia, Rio do Sul, SC, Brasil; 3 Universidade Federal do Paraná, Departamento de Saúde Coletiva, Curitiba, PR, Brasil

**Keywords:** Violencia, Personas Transgénero, Atención Primaria de Salud, Transfobia, Violence, Transgender People, Primary Health Care, Transphobia

## Abstract

**Objective:**

The study aims to describe the violence perceived by transvestigender people in Brazil, linking it to primary healthcare.

**Methods:**

Fourteen transvestigender people in Brazil were interviewed through 2 focus groups and 8 interviews, conducted between December 2021 and April 2022. Data analysis was performed using a phenomenological-hermeneutic approach.

**Results:**

The statements were categorized into the following themes: “the unintentional nature of violence”, “violence in health services” and “the family as a producer of violence”. These categories show that the phenomenon of violence extends beyond intentional acts directed at a person, as originally conceptualized by the World Health Organization.

**Conclusion:**

The transvestigender population experiences both intentional and unintentional violence, which impacts their physical and emotional well-being. Primary healthcare can serve as an ally in addressing these forms of violence by ensuring comprehensive care.

## Study contributions

**Main results:**

Violence against the transvestigender population can be intentional and unintentional. Unintentional violence occurs in various contexts, including health services and family relationships.

**Implications for services:**

Violence against the transvestigender population can be intentional and unintentional. Unintentional violence occurs in various contexts, including health services and family relationships.

**Perspectives:**

Primary health care, due to its broad reach and network-based configuration, has the potential to engage the community and promote changes in the gender apparatus that contribute to violent discourse against this population.

## RESUMEN

**Objetivo:**

El estudio tiene como objetivo describir la violencia percibida por personas transvestigénero en Brasil, articulándola con la atención primaria.

**Métodos:**

Se escuchó a catorce personas transvestigénero en Brasil a través de 2 grupos focales y 8 entrevistas, realizadas entre diciembre de 2021 y abril de 2022. El análisis de los datos utilizó un enfoque fenomenológicohermenéutico.

**Resultados:**

Las declaraciones se agruparon en los siguientes temas: “el carácter no intencional de la violencia”, “la violencia en los servicios de salud” y “la familia como productora de violencia”. Estas categorías evidencian que el fenómeno de la violencia va más allá de actos intencionales dirigidos a una persona, tal como fue originalmente concebido por la Organización Mundial de la Salud.

**Conclusión:**

La población transvestigénero experimenta violencias tanto intencionales como no intencionales que afectan su integridad física y emocional. La atención primaria de salud puede configurarse como una aliada en el enfrentamiento de estas violencias si garantiza la integralidad del cuidado.

## INTRODUCTION

Transvestigender people are those who fall outside the binary discourses of gender, encompassing transvestite, transgender and non-binary people.^1^ This population is not accounted for in the Brazilian census, which makes it impossible to measure mortality rates and other health indicators. Civil associations have dedicated efforts to producing data on the violence experienced by these people both globally, such as Transgender Europe,^2^ and in Brazil, such as the National Association of Transvestites and Transsexuals (*Associação Nacional de Travestis e Transexuais* - ANTRA).^3^

Violence targeting the transgender population in Brazil presents alarming data. Every year, on the National Transgender Day of Visibility (January 29), ANTRA publishes a dossier on murders and violence against the transvestigender population. The report published in 2024 identified a 10.7% increase in murders of transvestigender people in Brazil compared to 2022.^6^ It is worth highlighting that, during the same period, there was a 5.7% decrease in the overall murder rates in the Brazilian population.^4^

Violent deaths represent one of the biggest public health problems in the country, with significant impacts on society. These deaths also affect the victims’ families, making it a high-magnitude problem.^5^ Brazil remains the country with the highest number of homicides of transvestigender people,^6^ and the lack of official data hampers the development of effective public policies to combat violence against this population. The high widespread reach of the Brazilian National Health System (*Sistema Único de Saúde* – SUS), through primary healthcare centers, can be considered a potential ally in addressing the violence these people face in the country.

The institutionalization of the SUS resulted from the social reform that followed the Brazil’s military dictatorship between 1964 and 1985. Public health emerged as a field of action and power to rethink healthcare.^7^ This occurred after the VIII National Health Conference and in conjunction with discussions on the increase in inequality in the country and the international discussions raised by the Alma-Ata Conference on prioritizing the reduction of social inequalities and the criticism of fragmented biomedical knowledge. Universality, comprehensiveness and equity are among the principles of the SUS, shaping the values shared between health system users and workers. The guidelines of decentralization, hierarchy, regionalization and popular participation also inform the operationalization of the SUS.^8^

The principle of comprehensiveness is highlighted as one that, in one of its aspects, guides the clinical management of SUS users. In contrast to fragmented biomedical care, comprehensive care provides for assistance, prevention and health promotion. It requires the articulation of investigations based on the spontaneous demand of users to aid in prevention and health promotion.^9^ This articulation is understood through the hierarchy of healthcare levels.^10^ The articulation of the health system as a network is essential to achieving comprehensive care, as it dissolves hierarchical and fragmented logic, fostering shared responsibility and continuity of care.

Primary health care (PHC) is designed to resolve the population’s health issues, preventing higher levels of care from being overwhelmed by avoidable cases.^11^ Violence targeting the transvestigender population fits within this context. As a preventable health issue, violence should be addressed according to the principles of PHC. Given PHC’s role as the primary resource for prevention and health promotion, the National Primary Health Care Policy ( *Política Nacional de Atenção Básica* - PNAB) offers guidelines for healthcare professionals and managers to operationalize PHC.

This article aims to describe the violence perceived by transvestigender people in Brazil.

## METHODS

This study was a qualitative research project employing a hermeneutic-phenomenological approach, which used online focus groups and in-depth interviews as data collection methods. The study was conducted, entirely online, within the Postgraduate Program in Public Health at the Universidade Federal de Santa Catarina.

**Figure 1:**
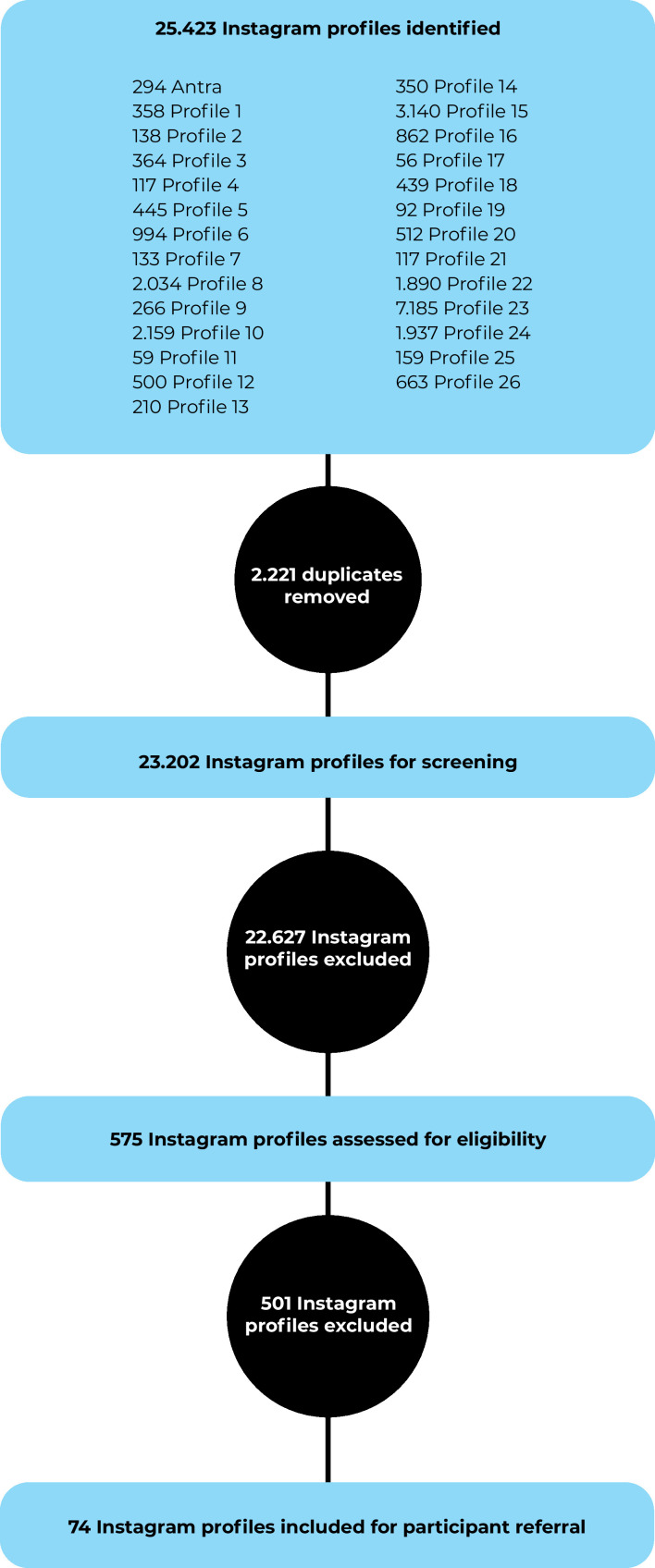
Flowchart of the mapping and screening process of contacted civil organizations

Participants were contacted via direct messages on Instagram and were referred by civil associations. To avoid invasive or harmful approaches, it was decided to receive referrals directly from associations that already had prior contact with these individuals. The mapping of civil associations was based on the analysis of the profiles followed by ANTRA on Instagram, with the aim of identifying which of these organizations worked directly with the transvestigender population. Following this initial mapping, data were extracted from 27 profiles using IGExport. The initial extraction resulted in 25,423 profiles. After removing duplicates, 22,627 profiles were excluded based on the analysis of the profile names, and subsequently, 501 profiles were excluded after content analysis. This left 74 civil association profiles for contact, as illustrated in Figure 1.

Based on these referrals, 24 individuals were scheduled to participate in focus groups or interviews. Of these, 14 effectively participated in the study: 5 transmasculine, 4 transgender women, 3 non-binary people and 2 transvestites. The age of the participants ranged from 21 to 29 years old. Regarding the place of residence, 1 person was from Ceará state; 1, from the Federal District; 4 were from Espírito Santo state; 1 was from Pernambuco state; 4 were from Rio de Janeiro state; 1 was from Rio Grande do Sul state; 1, from Santa Catarina state; and 1, from São Paulo state.

Data collection took place between December 2021 and April 2022, comprising 2 focus groups and 8 in-depth interviews. The duration of the focus groups was approximately 1 hour and 30 minutes, while the interviews lasted between 23 to 54 minutes.

During the study, the strategy of conducting focus groups was abandoned, as it was believed that the groups did not reach their full investigative potential. This was due to the fact that a key operation of focus groups is to stimulate participation from all involved, and with the cameras turned off, it was difficult to perceive the participants’ reactions to the conversation. In addition to the problems of unstable connection, one individual had recently been assaulted and did not turn on their camera, and it was not requested that they do so. It was concluded that, because violence is a sensitive topic, combined with the power dynamics between academia - which holds the knowledge - and the participants, who are often expelled from the academic environment,^12^ online interviews offered greater psychological protection and more flexibility in managing the conversation.

Despite this change, the data obtained from the focus groups were considered sufficient and were therefore included in the analysis. The assessment of the information richness is discussed by Martínez-Salgado,^13^ who suggests that the analysis of the data should consider the depth of the information, especially when articulated with the theoretical and epistemological framework of the research. The epistemological and theoretical basis of this study includes queer studies and postmodern epistemology, as well as decolonial and poststructuralist studies.

The sample size was determined by the “information power” contained in the participants’ responses. The data saturation strategy, which is common in qualitative research, was not followed. We agree with Martinez-Salgado,^13^ who suggests that the notion of data saturation implies that, at some point, the data become unusable, which is not consistent with the perspective adopted in this study. The author suggests that the sample should be selected based on the information richness and that the number of participants should be determined when there is sufficient material to deepen the research on the subject.^13^

All conversations were transcribed for the analysis. The analysis was performed based on the text produced from the transcription, without distinguishing between the data obtained from the focus groups and interviews. The data were organized through thematic analysis, which identified three categories: “the unintentional nature of violence”, “violence in health services” and “the family as a producer of violence”.

Hermeneutic phenomenology is an interpretative method that seeks to attribute meaning to the lived experiences of participants.^14^ This method is configured as a paradigm that assumes the impossibility of separating subject and object, in contrast to the positivist paradigm. Positivism, as an assumption of modern science, is hegemonic knowledge that establishes a causal link between phenomena, making them observable.^14,15^ For this study, critical epidemiology was used for data analysis. Priority was given to valuing participants’ statements over universalization based on the causal links common to the linear classical epidemiology, which attributes cause and effect to intrinsic and observable relationships between phenomena.^16^ It was believed that presenting the data based on the singularity of the statements, which dialogue with other studies, allows for an articulation with the principles of universality, comprehensiveness and equity of the SUS. This ensures that each individual is heard in their uniqueness, guaranteeing their access to healthcare.

The research was approved by the Research Ethics Committee of the Universidade Federal de Santa Catarina, opinion No. 5,033,418 dated 12/10/2021, certificate of submission for ethical appraisal No. 49713521.1.0000.0121. For participation in the study, online signing of the free and informed consent form was requested. For the presentation of the data, code names were used in order to preserve the identity of the participants.

## RESULTS

The participants’ statements in this study reveal the complexity in defining violence and highlight the presence of violent relationships targeting the transvestigender population. These relationships manifest within the family and health services.

### The unintentional nature of violence

The participants’ statements describe the complexity of the definition of violence.

They highlight the unintentional aspect of violence by mentioning that rejection, stares, and the lack of recognition of their identities in different contexts, such as in healthcare settings or in romantic relationships, also constitute forms of violence. Box 1 illustrates these perceptions, presenting statements from the participants when asked about what they consider violence. They point out the existence of both intentional and unintentional violence, the latter being exemplified by curious and rejection stares, or situations in which someone makes a pronoun mistake and becomes embarrassed by the error. The participants understand that, although the error may not be intentional, it reveals a failure to recognize who they are.

**Box 1:** Statements from study participants according to emerging themes

Theme	Speeches of the interlocutors
**The unintentional nature of violence**	“For me, violence encompasses different areas of life, not just the physical aspect, but even just a harsh look at me affects me feels like violence.” (Isabela, transgender woman, 35 years old).
	“There are forms of violence that some people don’t realize they are committing” (Ricardo, transmasculine person, 22 years old).
	“Sometimes the violence someone commits is unintentional, but once they realize, ‘Oh, this thing I did is transphobia, and it’s a kind of violence,’ they might stop, right? Well, it depends on the person, but sometimes they stop once they realize.” (Ailton, transmasculine person, 30 years old).
**The family as a producer of violence**	“I saw the family hugging and saying they loved each other, but I never felt it. Today I feel it. I know that my mother, the person who used to call me a freak back then, today she admires me, you know? Today I am married, building a family, everything I never had before, but I went through a lot to get here” (Isabela, trans woman, 35 years old).
	“We need love, you know? Society already pushes us away. It kicks us out of places, you know? So, if we don’t have this support network, whether it’s family or friends, we can’t live properly, you know, a lot of people end up giving up on life too, you know?” (Ricardo, transmasculine person, 22 years old).
	“When I was underage and didn’t have a job, my parents had a lot of control over me. They would say, ‘Oh, you’re a minor and you’re under my roof.’ That made things even more violent because I didn’t have the freedom to wear the clothes I was comfortable in. I didn’t stop cutting my hair until I turned 18, because I wasn’t free to have the haircut I liked..” (Diego, transmasculine person, 22 years old).
**Violence in health services**	“When I went to get my SUS card, I had so many issues. People didn’t know how to handle my social name. They’re not trained or ready to book appointments with a gynecologist. If your documents are already corrected, they can’t recognize you. Transmasculine people need access to a gynecologist, but they just can’t understand that. So here I am, all corrected, but I get referred to a neurologist instead of a gynecologist.” (Vagner, transmasculine person, 31 years old).
	“It is clear that health professionals contribute to the violence and exclusion these people face in society, which only worsens mental health problems” (Ricardo, transmasculine person, 22 years old).
	“Once you start hormone therapy, you stop being a whole individual. You’re just that person on hormone therapy now” (Benê, transmasculine person, 21 years old).
	“At a certain point during the exam, she turned to me and said: ‘if you were a man, you wouldn’t be here taking this exam’. And then it was completely embarrassing and humiliating, you know? Because that’s it, these are not rare situations, this is the norm” (Benê, transmasculine person, 21 years old).

### Violence in healthcare services

The interviewees described experiences in which they were often treated as having mental disorders or as hormone users, disregarding their complexities and individual identities. When these people have their documentation rectified, once registered in the system as male or female, these individuals are required to present physical attributes that are socially understood as feminine or masculine. There were reports of transmasculine people for whom the system did not allow referral for mammogram because, as this individual had already had their name and sex rectified, the system understood that people registered as male did not require mammography, assuming they are cisgender people. This participant, after much explanation to the health professional, was able to access the exam, but was subjected to a transphobic comment, as evidenced in Box 1.

Regarding the transgender outpatient clinics attended by participants, difficulties in access were reported, such as long waiting lines and even the lack of these services in their cities. Some reported outpatient clinics that offered endocrinological care, but did not have the necessary medications. The reports highlight the challenges faced in health services, including the frequent lack of use of the correct name and pronouns.

A recurring difficulty among healthcare professionals was noted in recognizing the participants’ needs beyond mental health care. The reports suggest that there is a common assumption that these individuals only require mental health services, neglecting other types of care.

### The family as a producer of violence

The participants identified family and social support networks as fundamental in coping with violence. Many emphasize that access to information is crucial in reducing transphobia. Some mentioned that they found a sense of family with brothel madams, as sex work was what initially provided them shelter.

Among the participants, there are cases where the family exerts control over the bodies of underage youth, preventing them from earning their own money for survival and forcing them to remain within the family environment.

The lack of recognition by the family as a valid possibility for existence was reported by one participant, who chose to leave home to avoid continuing to suffer familial violence. This individual felt deprived of the right to be who they aspired to become, a choice they associated with the destruction of their life, leading to substance abuse. In this case, this participant was only able to leave what they called the “underworld” after getting a formal employment.

Those who did not experience family rejection highlighted the importance of family in helping them cope with the lack of societal recognition of their bodies, underscoring the crucial role of support networks.

## DISCUSSION

Data from this research highlight the complexity of the violence experienced by transvestigender people, encompassing various dimensions of their lives, and shows that it does not always result from intentional action, as pointed out by the World Health Organization (WHO).^17^ The WHO defines violence as: “The intentional use of physical force or power, threatened or actual, against oneself, another person, or against a group or community, that either results in or has a high likelihood of resulting in injury, death, psychological harm, developmental disability, or deprivation.”^17^

It could be seen that the data presented here indicate that violence is configured by the erasure of the victim’s subjectivity, imposed by the other, that is, by the lack of legitimacy of the subject’s unique characteristics. This may relate to physical body traits, the lack of recognition of the person’s self-declared identity, or, in more extreme cases, result in aggression or even the extermination of a body perceived as unintelligible, as defined by Judith Butler.^18-20^

The complexity of transvestigender identities arises from their positioning within the apparatus of sexuality,^20, 21^ and are treated as abject identities. Butler^18^ defines the abject as that which is excluded during the constitutive process of subjectivity, creating uninhabitable zones and identities that do not achieve the status of subject. It is understood that the social discourse that creates and perpetuates abject identities, when legitimized and named by the State, transcends abjection, constituting transphobia.^22^ Jorge Leite Júnior^23^ will highlight the monstrous character that these people acquire when situated within the realm of the abject. Recalling the Western notion of the monster–one that simultaneously elicits curiosity and disgust and is something that one desires to exterminate–the monster is the intelligible, and this is the aspect these individuals acquire in the eyes of non-intelligibility.

This research shows that these bodies become unintelligible when they enter the healthcare system, since biomedical knowledge guides health services. This affects how these individuals are considered, reducing their subjectivities. In this context, binary notions emerge, and the concept of continuity, as described by Butler,^19^ is reaffirmed.

Thus, gaps in PHC are perceived when it comes to ensuring the right to health for transvestigender people, particularly regarding the principle of comprehensiveness and the guidelines established by the PNAB.^11^ This happens because, as pointed out by Bousquat et al.,^10^ PHC is responsible for providing resolutive and comprehensive care to achieve universal access to healthcare. The authors also highlight, as was observed in this study, the challenges in coordinating health care networks in Brazil. Regarding health care for the transvestite population, Santos^24^ underscores the need for more active involvement from the Ministry of Health to address the specific demands of this population. In addition to the services provided, this population demands access to body modifications, and, as observed in this study, there is still a gap in access to hormone therapy and surgeries within the SUS transsexualization process.

An analysis of the participants’ statements reveals that policies that should be well-established within PHC, such as respect for the social name of users, are not yet standard practice among healthcare professionals. Ordinance No. 1,820, of August 13, 2009,^25^ guarantees the rights of healthcare users and ensures the use of social names and prejudice-free reception. Nevertheless, there are still reports of transfobia resulting from non-comprehensive and transphobic care, rooted in a binary male/female logic, as problematized by queer studies.

It is essential to consider how PHC and PNAB can play a role in addressing violence against these bodies, not only at the level of care, but also at the discursive level, seeking to prevent and promote health. Decentralization, a guiding principle of the PNAB,^11^ is highlighted for its role in expanding the reach of healthcare facilities, making the SUS more accessible across Brazil’s more than 5,000 municipalities. Decentralization can be seen as a strategy to distribute decision-making power on health policies, aligning them with population demands.^8^ In conjunction with the principle of comprehensiveness, these guidelines can rely on equity to guarantee universal access to health for transvestigender people. This is justified, as Mattos points out, because “defending comprehensiveness means defending, above all, that health practices in the SUS are always intersubjective, in which healthcare professionals engage subjects, not objects”.^9^

Comprehensive care, which includes health promotion and prevention, offers an alternative to the fragmented, biomedical, and assistance-oriented approach still prevalent in many healthcare training programs. The importance of training health professionals to care for and welcome the transvestigender population is highlighted, an area that still presents significant gaps. Studies on curricular guidelines in healthcare programs point to a gap in the critical approach to gender identities and sexuality.^26, 27^

The eMulti^28^ stands out as a public policy aligned with the proposal of this study, as it holds the community accountable for improving the resolution of health issues, such as violence against the transvestigender and LGBTQIAPN+ population (lesbians, gays, bisexuals, transsexuals, queer/questioning, intersex, asexual/aromantic/agender, pansexual/polysexuals, non-binary, and others). The logic of co-responsibility allows health professionals, when working with the community, to discuss the violence present in family relationships, schools, community centers, and religious institutions. Healthcare services can encourage the construction of new discourses around the transvestigender population, challenging and dispelling the strangeness and monstrosity inherent in binary social logics. The ability of PHC to provide hormone distribution and local follow-up is also crucial. This prevents the need for travel to specialized outpatient clinics located only in large urban centers, which often have long waiting lines.

Comprehensive care, combined with networked collaboration, positions PHC as a potential ally in conducting cross-sectoral work with the transvestigender population. This approach is essential to raise awareness and address the violence that these people face in society. A more proactive and focused engagement with the transvestigender population within PHC is vital to moving beyond dichotomous biomedical logics and allowing transvestigender identities to exist outside the realm of abject monstrosity.

Among the limitations of this research, it is worth highlighting that it was conducted exclusively by cisgender people. This created challenges in accessing the field, given the vulnerability of transvestigender people in the context of transphobia socially imposed by cisgender people. This dynamic may have been reinforced by power structures that intersect both social class and the researcher-participant relationship. This limited the depth of the inquiry, as the study relied on online interviews and focus groups as data collection tools. Although the online format was useful because it allowed the research to reach more people than would have been possible in person, it also limited the depth of the issues addressed. This was partly due to the difficulty in observing signs of comfort and discomfort during interactions, which led to the avoidance of exposing participants to possible violence. Consequently, some areas were not explored in greater depth.

In conclusion, violence against transvestigender people must be understood not only in its intentional aspect, but also in its unintentional forms. Addressing this reality requires that the principle of comprehensiveness be effectively implemented in healthcare services, with special attention to primary healthcare.

## References

[1] Silva FGO (2022). Perspectivas de um campo de pesquisa travesti-transexual-transgênero ou transvestigenere. Koan: Revista de Educação e Complexidade.

[2] Transgender Europe (TGEU) (2024). Trans murder monitoring.

[3] Associação Nacional de Travestis e Transexuais (Antra) (2024). Assassinatos: pesquisas anuais.

[4] Benevides BG (2024). Dossiê: Assassinatos e violências contra travestis e transexuais brasileiras em 2023.

[5] Pimenta FG (2007). Violência: prevenção e controle no Brasil. Epidemiol Serv Saude.

[6] Transgender Europe (TGEU) (2023). Trans murder monitoring update: Trans Day of Remembrance 2023.

[7] Paiva CHA, Teixeira LA (2014). Reforma sanitária e a criação do Sistema Único de Saúde: notas sobre contextos e autores. Hist Ciênc Saúde-Manguinhos.

[8] Matta GC (2007). Políticas de saúde: organização e operacionalização do Sistema Único de Saúde.

[9] Mattos RA (2004). A integralidade na prática (ou sobre a prática da integralidade). Cad Saúde Pública.

[10] Bousquat A, Giovanella L, Fausto MCR, Medina MG, Martins CL, Almeida PF (2019). A atenção primária em regiões de saúde: política, estrutura e organização. Cad Saúde Pública.

[11] Brasil (2017). Portaria nº 2.436, de 21 de setembro de 2017. Aprova a Política Nacional de Atenção Básica, estabelecendo a revisão de diretrizes para a organização da Atenção Básica, no âmbito do Sistema Único de Saúde (SUS).

[12] Souza MHT (2013). Itinerários terapêuticos das travestis de Santa Maria/RS.

[13] Martínez-Salgado C (2021). Tópicos avançados em pesquisa qualitativa em saúde: fundamentos teórico-metodológicos.

[14] Gastaldo DM (2021). Tópicos avançados em pesquisa qualitativa em saúde: fundamentos teórico-metodológicos.

[15] Chauí MS (1987). Primeira Filosofia: aspectos da história da filosofia.

[16] Breilh J (2013). La determinación social de la salud como herramienta de transformación hacia una nueva salud pública. Rev Fac Nac Salud Pública.

[17] Organização Mundial da Saúde (OMS) (2002). Relatório mundial sobre violência e saúde.

[18] Butler J (2019). Corpos que importam: os limites discursivos do “sexo”.

[19] Butler J (2013). Problemas de gênero: feminismo e subversão da identidade.

[20] Foucault M (2015). A História da Sexualidade I: A Vontade de Saber.

[21] Bento B (2017). A Reinvenção do Corpo: sexualidade e gênero na experiência transexual.

[22] Koltai C (2008). Racismo: uma questão cada vez mais delicada. Ide.

[23] Leite J (2012). Transitar para onde? Monstruosidade, (des)patologização, (in)segurança social e identidades transgêneras. Rev Estud Fem.

[24] Santos MCB (2020). Aos trancos e barrancos: uma análise do processo de implementação e capilarização do processo transexualizador no Brasil.

[25] Brasil (2009). Portaria GM/MS n. 1.820, de 13 de agosto de 2009. Dispõe sobre os direitos e deveres dos usuários da saúde.

[26] Medeiros ES, Oliveira JB, Leiria M, Moretti-Pires RO, Mello MMC (2023). A formação de estudantes de Medicina para o cuidado destinado à saúde de pessoas LGBTI+. Rev Bras Educ Med.

[27] Popadiuk GS, Oliveira DC, Signorelli MC (2017). A política nacional de saúde integral de lésbicas, gays, bissexuais e transgêneros (LGBT) e o acesso ao processo transexualizador no Sistema Único de Saúde (SUS): Avanços e desafios. Ciênc Saúde Colet.

[28] Brasil (2023). Portaria GM/MS n. 635, de 22 de maio de 2023. Institui, define e cria incentivo financeiro federal de implantação, custeio e desempenho para as modalidades de equipes Multiprofissionais na Atenção Primária à Saúde.

