# Supplementary material for: Perception of violence by transvestigender people in Brazil and its implications for primary health care
Source: Epidemiol Serv Saude. 2025 Jan 10;33(spe1):e2024283. doi: 10.1590/S2237-96222024v33e2024283.especial.en (PMC11734658; doi:10.1590/S2237-96222024v33e2024283.especial.en)
Supplement: Translation

# Translation: A percepção da violência por pessoas transvestigêneres no Brasil e suas implicações na atenção primária à saúde

**DOI:** 10.1590/S2237-96222024v33e2024283.especial.pt

**Authors:** Mônica Machado Cunha e Mello, Rodrigo Otávio Moretti-Pires, Marcos Claudio Signorelli

**Affiliations:** 1 Universidade Federal de Santa Catarina, Programa de Pós-Graduação em Saúde Coletiva, Florianópolis, SC, Brasil; 2 Centro Universitário para o Desenvolvimento do Alto Vale do Itajaí, Curso de Psicologia, Rio do Sul, SC, Brasil; 3 Universidade Federal do Paraná, Departamento de Saúde Coletiva, Curitiba, PR, Brasil

**Keywords:** Violência, Pessoas Transgênero, Atenção Primária à Saúde, Transfobia

## Abstract

**Objetivo:**

O estudo tem como objetivo descrever a violência percebida por pessoas transvestigêneres no Brasil, articulando-a com a atenção primária.

**Métodos:**

Catorze pessoas transvestigêneres no Brasil foram ouvidas por meio de 2 grupos focais e 8 entrevistas, realizadas entre dezembro de 2021 e abril de 2022. A análise dos dados utilizou uma abordagem fenomenológica-hermenêutica.

**Resultados:**

As falas foram agrupadas nos seguintes temas: “o caráter não intencional da violência”, “a violência nos serviços de saúde” e “a família enquanto produtora de violência”. Essas categorias evidenciam que o fenômeno da violência vai além de atos intencionais direcionados a uma pessoa, conforme originalmente pensado pela Organização Mundial da Saúde.

**Conclusão:**

A população transvestigênere experiencia violências intencionais e não intencionais que afetam sua integridade física e emocional. A atenção primária à saúde pode se configurar como uma aliada no enfrentamento dessas violências se garantir a integralidade do cuidado.

## Contribuições do estudo

**Principais resultados:**

A violência contra a população transvestigênere pode ser intencional e não intencional. A violência não intencional está presente em diversas dimensões como nos serviços de saúde e nas relações familiares.

**Implicações para os serviços:**

A atenção primária à saúde, devido à sua capilaridade e à configuração de trabalho em rede, tem potencial alcance na comunidade para promover mudanças no dispositivo de gênero que provoca um discurso violento em relação a essa população.

**Perspectivas:**

É importante compreender o combate à violência contra a população transvestigênere como uma transformação discursiva social. É necessário um trabalho transversal entre profissionais de saúde e a comunidade, envolvendo uma corresponsabilização.

## INTRODUÇÃO

Pessoas transvestigêneres são aquelas que se situam fora das discursividades binárias de gênero, abarcando as pessoas travestis, transgêneros e não binários.^1^ Essa população não é contabilizada pelo censo brasileiro, o que inviabiliza a mensuração de taxas de mortalidade e outros indicadores de saúde. Associações civis têm se dedicado à produção de dados sobre a violência sofrida por essas pessoas tanto em âmbito global, como a Transgender Europe,^2^ quanto no Brasil, como a Associação Nacional de Travestis e Transexuais (Antra).^3^

A violência direcionada à população transvestigênere no Brasil apresenta dados alarmantes. Anualmente a Antra publica, no Dia Nacional da Visibilidade Trans (29 de janeiro), um dossiê sobre assassinatos e violências contra a população transvestigênere. O relatório publicado em 2024 identificou um aumento de 10,7% nos assassinatos de pessoas transvestigêneres no Brasil em comparação a 2022.^6^ É importante notar que, no mesmo período, houve queda de 5,7% nos assassinatos gerais na população brasileira.^4^

Mortes decorrentes de violência representam um dos maiores problemas de saúde pública do país, com grandes impactos na sociedade. Essas mortes também afetam os familiares das vítimas, configurando-se como um problema de alta magnitude.^5^ O Brasil permanece como o país com o maior número de homicídios de pessoas transvestigêneres,^6^ e a falta de dados oficiais impede o desenvolvimento de políticas públicas eficazes para combater a violência contra essa população. A alta capilarização do Sistema Único de Saúde (SUS), por meio das unidades básicas de saúde, pode ser pensada como potencial aliada na atuação contra a violência que essas pessoas sofrem no país. 

A institucionalização do SUS resultou da reforma social no final do período de ditadura militar que o Brasil enfrentou entre 1964 e 1985. A saúde coletiva emergiu como um campo de atuação e poder para reconstruir o pensamento em saúde.^7^ Isso ocorreu a partir da VIII Conferência de Saúde e em conjunto com as discussões sobre o aumento da desigualdade no país e as discussões internacionais levantadas pela Conferência de Alma-Ata sobre a priorização da redução de desigualdades sociais e a crítica ao saber biomédico fragmentado. A universalidade, a integralidade e a equidade estão entre os princípios do SUS. Estes orientam os valores a serem seguidos entre os cidadãos usuários e os trabalhadores da saúde. As diretrizes de descentralização, hierarquização e regionalização e a participação popular também orientam a operacionalização do SUS.^8^

O princípio de integralidade é destacado como aquele que, em um dos seus aspectos, norteia a condução clínica dos usuários do SUS. Em contraste com o assistencialismo biomédico fragmentado, a integralidade do cuidado prevê a assistência, a prevenção e a promoção à saúde. É necessário articular, a partir de uma demanda espontânea dos usuários, investigações que auxiliem na prevenção e promoção da saúde.^9^ Na prática, essa articulação pode ser compreendida a partir da hierarquização da atenção em níveis de atenção.^10^ É essencial a articulação do sistema de saúde em rede para que a lógica da integralidade se efetive, uma vez que a rede permite que a lógica hierárquica e fragmentada se dissolva, dando lugar à corresponsabilização e à longitudinalidade do cuidado.

A atenção primária à saúde (APS) prevê a resolubilidade dos casos de adoecimento da população, poupando os níveis mais altos de atenção de demandas evitáveis.^11^ A violência direcionada à população transvestigênere se enquadra nesse contexto. Enquanto agravo de saúde evitável, a violência deve ser abordada conforme os princípios da APS. Considerando a APS como o principal recurso para a prevenção e promoção da saúde, a Política Nacional de Atenção Básica (PNAB) oferece diretrizes que orientam os profissionais de saúde e gestores na operacionalização da APS.

Este artigo tem como objetivo descrever a violência percebida por pessoas transvestigêneres no Brasil.

## MÉTODOS

Este estudo é uma pesquisa qualitativa com abordagem fenomenológico-hermenêutica, que utilizou grupos focais onlinee entrevistas em profundidade como métodos de coleta de dados. O estudo foi realizado no Programa de Pós-Graduação em Saúde Coletiva da Universidade Federal de Santa Catarina e conduzido inteiramente de forma *online*.

Contatadas por meio de mensagens diretas no Instagram, as pessoas participantes da pesquisa foram indicadas por associações civis. Para evitar abordagens invasivas ou violentas, optou-se por receber as indicações diretamente de associações que já mantinham contato prévio com essas pessoas. O mapeamento das associações civis foi realizado a partir da análise dos perfis seguidos pela Antra no Instagram, com o objetivo de identificar quais dessas organizações atuavam diretamente com a população transvestigênere. Após esse primeiro mapeamento, extraíram-se os dados de 27 perfis com uso da IGExport. A extração inicial resultou em 25.423 perfis. Após a remoção das duplicatas, 22.627 perfis foram excluídos com base na análise dos nomes dos perfis, e, em seguida, 501 perfis foram excluídos após a análise do conteúdo das contas. Restaram 74 perfis de associações civis para contato. Esse processo está ilustrado na Figura 1.

**Figura 1:** Esquema do processo de mapeamento e triagem das organizações civis contatadas

A partir dessas indicações, foi possível agendar a participação de 24 pessoas nos grupos focais ou entrevistas. Dessas, 14 participaram efetivamente do estudo: 5 transmasculinas, 4 mulheres trans, 3 não binárias e 2 travestis. A idade das pessoas participantes variou entre 21 e 29 anos. Em relação ao estado de residência, 1 pessoa era do Ceará; 1, do Distrito Federal; 4 eram do Espírito Santo; 1 era de Pernambuco; 4 eram do Rio de Janeiro; 1 era do Rio Grande do Sul; 1, de Santa Catarina; e 1, de São Paulo.

A coleta de dados ocorreu entre dezembro de 2021 e abril de 2022, abrangendo 2 grupos focais e 8 entrevistas em profundidade. A duração dos grupos focaisfoi aproximadamente 1h30, enquanto as entrevistas variaram de 23 a 54 minutos. 

Durante a condução do estudo, abandonou-se a estratégia de realização dos grupos focais, pois se acreditou que os grupos não alcançaram sua potencialidade investigativa. Isso ocorreu porque uma operação essencial para um grupo focal é estimular a participação de todos os envolvidos, e, com as câmeras desligadas, foi difícil perceber as reações dos participantes à conversa. Além dos problemas de instabilidade na conexão, houve o caso de uma pessoa recém-agredida que não abriu a câmera, e não foi pedido que o fizesse. Concluiu-se que, por se tratar de um tema sensível como a violência, aliado às dinâmicas de poder entre a academia ‒ que detém o conhecimento ‒ e as pessoas participantes, frequentemente expulsas do ambiente acadêmico,^12^ as entrevistas online ofereciam maior garantia de resguardo psicológico e maior flexibilidade no manejo da conversa.

Embora tenha havido essa mudança, os dados obtidos nos grupos focais foram considerados suficientes e, portanto, incluídos na análise. A avaliação da riqueza das informações coletadas é discutida por Martínez-Salgado,^13^ a qual sugere que a análise dos dados deve considerar a profundidade das informações, especialmente quando articulada com a base teórica e epistemológica da pesquisa. A base epistemológica e teórica deste estudo inclui os estudos queer e a epistemologia pós-moderna, além dos estudos decoloniais e pós-estruturalistas.

O tamanho da amostra foi determinado pelo “poder de informação” contido nas respostas das pessoas interlocutoras. Não se seguiu a estratégia de saturação de dados, que é comum em pesquisas qualitativas. Concorda-se com Martinez-Salgado,^13^ a qual sugere que a noção de saturação de dados implica que, em algum momento, os dados se tornariam inutilizáveis, o que não condiz com a perspectiva adotada neste estudo. A autora sugere que a amostragem seja selecionada com base na riqueza de informações obtidas e que o número de participantes seja determinado quando houver material rico o suficiente para aprofundar a pesquisa do tema.^13^

Para a análise, todas as conversas foram transcritas. O trabalho de análise foi realizado com base no texto produzido pela transcrição, sem distinguir os dados obtidos pelos grupos focais e entrevistas. Os dados foram organizados por meio de uma análise temática, que identificou três categorias: “o caráter não intencional da violência”, “a violência nos serviços de saúde” e “a família enquanto produtora de violência”.

A fenomenologia hermenêutica é um método interpretativo que busca atribuir significado às experiências vividas pelos participantes.^14^ Esse método se configura como um paradigma que pressupõe a impossibilidade de separação entre sujeito e objeto, em contraste com o paradigma positivista. O positivismo, como pressuposto da ciência moderna, é um saber hegemônico que estabelece uma noção de nexo causal entre os fenômenos, tornando-os passíveis de observação.^14,15^ Para este estudo, optou-se pela epidemiologia crítica na análise dos dados. Foi priorizada a valorização das falas das pessoas participantes em vez de uma universalização fundamentada no nexo causal comum nas quantificações da epidemiologia clássica linear, que atribui causa e efeito a relações intrínsecas e observáveis dos fenômenos.^16^ Acreditou-se que a apresentação dos dados a partir da singularidade das falas, que dialogam com outros estudos, permite uma articulação com os princípios da universalidade, da integralidade e da equidade do SUS. Isso assegura que cada pessoa seja ouvida em sua singularidade, garantindo seu acesso à saúde.

A pesquisa foi aprovada pelo Comitê de ética em pesquisa com seres humanos da Universidade Federal de Santa Catarina por meio do parecer nº 5.033.418 de 12/10/2021, certificado de apresentação de apreciação ética nº 49713521.1.0000.0121. Para a participação na pesquisa, solicitou-se a assinatura online do termo de consentimento livre e esclarecido. Para a apresentação dos dados, foram utilizados codinomes com o objetivo de preservar a identidade dos participantes.

## RESULTADOS

As falas das pessoas participantes do estudo revelam a complexidade na definição da violência e evidenciam a presença de relações violentas direcionadas à população transvestigênere. Essas relações manifestam-se no âmbito familiar e nos serviços de saúde.

### O caráter não intencional da violência

As falas das pessoas interlocutoras descrevem a complexidade da definição de violência.

As pessoas interlocutoras destacam o aspecto não intencional da violência ao mencionarem que a rejeição, os olhares e o não reconhecimento de suas identidades em diferentes contextos, como nos espaços de saúde ou em relacionamentos amorosos, também configuram formas de violência. O Quadro 1 ilustra essas percepções, apresentando falas das interlocutoras quando questionadas sobre o que consideram violência. Elas apontam a existência de violências intencionais e não intencionais, sendo estas últimas exemplificadas por olhares de curiosidade e rejeição, ou situações em que alguém erra o pronome e se constrange pelo erro. As pessoas interlocutoras entendem que, embora o erro possa não ser intencional, ele revela uma falha no reconhecimento de quem elas são.

**Quadro 1:** Falas das pessoas participantes da pesquisa, de acordo com os temas emergentes

Tema	Falas das pessoas interlocutoras
**O caráter não intencional da violência**	“A violência para mim abrange diversas áreas da vida, né, não só a questão física, mas até um olhar violento para mim me atinge como violência, sim” (Isabela, mulher trans, 35 anos).
	“Tem formas de violência que certas pessoas não percebem que elas estão cometendo essa violência” (Ricardo, pessoa transmasculina, 22 anos).
	“Muitas vezes essa violência que a pessoa cometia que era não intencional, a partir do momento que a pessoa tem esse estalo, de que ‘Ah, isso aqui que eu estou cometendo uma transfobia é um tipo de violência’, a partir daí a pessoa já começa, né, então já que tem nome eu vou parar, né, dependendo da pessoa, né” (Ailton, pessoa transmasculina, 30 anos).
**A família enquanto produtora** **de violência**	“Eu via a família se abraçando e dizendo que se amava, mas eu nunca senti. Hoje eu sinto. Eu sei que minha mãe, aquela pessoa que me chamou de aberração lá atrás, hoje ela me admira, você entendeu? Hoje eu sou casada, eu estou construindo uma família, tudo que eu nunca tive lá atrás, mas eu passei por muita coisa para chegar até aqui” (Isabela, mulher trans, 35 anos).
	“A gente precisa de amor, entendeu? A sociedade já afasta gente. Ela tira a gente dos lugares, entendeu? Então, se a gente não tem essa rede de apoio, seja de familiar, amigo, a gente acaba não conseguindo viver direito, sabe, muita gente acaba desistindo também da vida, entendeu?” (Ricardo, pessoa transmasculina, 22 anos).
	“Quando eu era menor de idade e não tinha trabalho, meus pais tinham muito controle sobre mim. Eles falavam: ‘ah, você é menor de idade e está debaixo do meu teto’. Isso acaba sendo violência maior, porque eu não tinha liberdade de usar a roupa que eu me sentia confortável usando. Eu fui parar de cortar o cabelo só com 18 anos, porque eu não tinha liberdade para ter o corte de cabelo que eu me sentia confortável” (Diego, pessoa transmasculina, 22 anos).
**A violência nos serviços de saúde**	“Quando eu fui fazer a carteirinha do SUS, eu tive muito problema. A pessoa não sabia como colocar o nome social, eles não têm formação, preparação as vezes para lidar e marcar ginecologista. Se você já tem a documentação retificada, as pessoas não conseguem te reconhecer. São pessoas transmasculinas que precisam ter o acesso ao ginecologista. Eles não conseguem entender isso. Então eu sou uma pessoa retificável, eu não sou passável, eu sou passado para neuro, mas não sou passado para um ginecologista” (Vagner, pessoa transmasculina, 31 anos).
	“Percebe-se que os profissionais de saúde contribuem para a violência e exclusão dessas pessoas na sociedade, favorecendo o desencadeamento de consequências mais graves para a saúde mental” (Ricardo, pessoa transmasculina, 22 anos).
	“A partir do momento que você começa um tratamento hormonal, você deixa de ser um indivíduo plural, você é só uma pessoa ali que está fazendo um tratamento” (Benê, pessoa transmasculina, 21 anos).
	“Em determinado momento do exame, ela virou, olhou para mim e falou: ‘se você fosse homem, você não estava aqui fazendo esse exame’. E aí foi completamente constrangedor e vexatório, sabe? Porque é isso, não são situações raras, esse é o padrão” (Benê, pessoa transmasculina, 21 anos).

### A violência nos serviços de saúde

As pessoas entrevistadas descrevem experiências nas quais elas são frequentemente tratadas como portadoras de transtornos mentais ou como usuárias de hormônios, desconsiderando suas complexidades e identidades individuais. Quando essas pessoas têm sua documentação retificada, uma vez registrada no sistema com um sexo masculino ou feminino, será necessário que essa pessoa apresente atributos físicos compreendidos socialmente como femininos ou masculinos. Houve relatos de pessoas transmasculinas em que o sistema não permitia o encaminhamento para exame de mamografia, pois, como essa pessoa já havia realizado a retificação de nome e sexo, o sistema compreendeu que pessoas registradas com sexo masculino não têm indicação para a realização de mamografia, por se tratar de pessoas cisgênero. Esse participante, após muita explicação ao profissional de saúde, conseguiu acessar o exame, mas sofreu uma fala transfóbica, como evidenciado no Quadro 1.

No que se refere aos ambulatórios trans frequentados pelos participantes, foram relatadas dificuldades de acesso, como as longas filas de espera e até mesmo a inexistência desses serviços em suas cidades. Há relatos de ambulatórios que oferecem acompanhamento médico endocrinológico, mas não dispõem dos medicamentos necessários. Os relatos destacam as dificuldades enfrentadas nos serviços de saúde, incluindo a frequente falta do uso do nome e do pronome corretos.

Constatou-se uma dificuldade recorrente por parte dos profissionais de saúde em reconhecer a Os relatos apontam que é comum a presunção de que essas pessoas precisam apenas de atendimento em saúde mental, em detrimento de outros tipos de cuidados.

### A família enquanto produtora de violência

As pessoas interlocutoras apontam a rede de apoio familiar e social como fundamental para enfrentar a violência. Muitos destacam que a informação é crucial para reduzir as transfobias. Algumas pessoas mencionaram que encontraram nas cafetinas uma possibilidade de família, pois foi a prostituição que as acolheu. 

Entre os participantes, há casos em que a família exerce controle sobre o corpo de jovens menores de idade, que não podem ganhar seu próprio dinheiro para a subsistência, obrigando-os a permanecer no ambiente familiar. 

A falta de reconhecimento da família como uma possibilidade de existência foi relatada por uma pessoa que escolheu sair de casa para evitar continuar sofrendo a violência familiar e se sentiu privada do direito de ser quem almejava. Essa escolha foi associada à destruição de sua vida, levando-a ao uso abusivo de drogas. Nesse caso, essa interlocutora só conseguiu sair do que denominou “submundo” após obter um trabalho formal.

As pessoas que não mencionaram rejeição familiar destacaram a importância que a família teve para que pudessem enfrentar o não reconhecimento social de seus corpos, evidenciando o papel crucial da rede de apoio.

## DISCUSSÃO

Os dados desta pesquisa evidenciam a complexidade da violência enfrentada pelas pessoas transvestigêneres, abrangendo diversas dimensões da vida dessas pessoas, e mostram que ela nem sempre resulta de ação intencional, como apontado pela Organização Mundial da Saúde (OMS).^17^A OMS define a violência como: “O uso intencional da força física ou do poder, real ou em ameaça, contra si próprio, contra outra pessoa, ou contra um grupo ou comunidade, que resulte ou tenha grande possibilidade de resultar em lesão, morte, dano psicológico, deficiência de desenvolvimento ou privação.”^17^

Compreende-se que os dados apresentados indicam que a violência se configura pelo apagamento da subjetividade que o outro impõe à vítima, ou seja, pela falta de legitimação das particularidades do sujeito. Isso pode ocorrer em relação aos traços físicos corporais, na ausência de reconhecimento da identidade autodeclarada pela pessoa, ou, em casos mais extremos, resultar em agressões ou até mesmo no extermínio de um corpo compreendido como não inteligível, conforme definido por Judith Butler.^18-20^

A complexidade das identidades transvestigêneres decorre de sua inserção no dispositivo da sexualidade,^20,21^ sendo tratadas como identidades abjetas. Butler^18^ define o abjeto como aquilo que é excluído durante o processo constitutivo da subjetividade, criando zonas inabitáveis e identidades que não alcançam o estatuto de sujeito. Entende-se que o discurso social que cria e perpetua identidades abjetas, quando legitimadas e nomeadas pelo Estado, transcende a abjeção, configurando-se como transfobia.^22^ Jorge Leite Júnior^23^ apontará o caráter de monstruosidade que essas pessoas adquirem quando inseridas no âmbito do abjeto. Retomando a noção ocidental de monstro ‒ aquele que, ao mesmo tempo, suscita curiosidade e nojo, e que se deseja exterminar ‒, o monstro é o inteligível, e esse aspecto é o que essas pessoas adquirem aos olhos da não inteligibilidade.

Essa pesquisa aponta que esses corpos tornam-se ininteligíveis quando adentram o sistema de saúde uma vez que o saber biomédico orienta os serviços de saúde, afetando-os ao considerá-los de maneira reduzida em suas subjetividades. Nesse contexto, a binaridade se apresenta, e a noção de continuidade, conforme descrita por Butler,^19^ se confirma.

Assim, percebe-se lacunas na APS para a efetivação do direito à saúde das pessoas transvestigêneres, em relação ao princípio da integralidade e às diretrizes estabelecidas pela PNAB.^11^ Isso ocorre porque, como apontam Bousquat et al.,^10^ cabe à APS configuração que seja resolutiva e integral para alcançar a universalidade do acesso à saúde. Os autores também destacam, assim como foi percebido neste estudo, os desafios para a articulação das redes de atenção à saúde no Brasil. No que se refere à atenção à saúde da população transvestigênere, Santos^24^ aponta para a necessidade de atuação mais intensa do Ministério da Saúde para atender às demandas dessa população. Além dos serviços previstos, essa população demanda acesso a modificações corporais, e, conforme foi observado neste estudo, ainda há defasagem no acesso à hormonização e às cirurgias no âmbito do processo transexualizador do SUS.

Ao analisar as falas das pessoas interlocutoras, observa-se que normativa que deveria estar bem estabelecida na APS, a do respeito ao nome social dos usuários, ainda não se configura como prática adotada pelos profissionais de saúde como realidade. A Portaria nº 1.820, de 13 de agosto de 2009,^25^ prevê os direitos dos usuários dos serviços de saúde e garante a utilização do nome social e o acolhimento livre de preconceitos. Existem ainda relatos das transfobias, como resultado de um atendimento não integral e transfóbico, centrado em lógica binária homem/mulher como problematizado pelos estudos *queer*. 

Deve ser ponderado como a APS e a PNAB podem atuar no enfrentamento da violência contra esses corpos, não apenas no nível assistencial, mas também no discursivo, buscando prevenir e promover a saúde. Destaca-se que a descentralização, como diretriz norteadora da PNAB,^11^ promove a capilaridade dos postos de saúde e facilita a interiorização do SUS em um país como o Brasil, que possui mais de 5 mil municípios. A descentralização pode ser vista como estratégia para distribuir o poder na tomada de decisões sobre políticas de saúde, alinhando-as às demandas populacionais.^8^ Em articulação com o princípio da integralidade, essas diretrizes podem recorrer à equidade para garantir a universalidade do acesso à saúde para pessoas transvestigêneres. Isso se justifica, como destaca Mattos, porque “defender a integralidade é defender, antes de tudo, que as práticas em saúde no SUS sejam sempre intersubjetivas, nas quais profissionais de saúde se relacionem com sujeitos, e não com objetos”.^9^

A integralidade do cuidado, ao incorporar promoção e prevenção à saúde, representa alternativa à abordagem biomédica fragmentada e assistencialista ainda predominante em muitos cursos de formação na área da saúde. Destaca-se a importância da formação dos profissionais de saúde para o atendimento e acolhimento da população transvestigênere, área que ainda apresenta defasagens significativas. Estudos sobre as diretrizes curriculares em cursos da área da saúde apontam para lacuna na abordagem crítica das identidades de gênero e da temática da sexualidade.^26,27^

Destaca-se o eMulti^28^ como política pública alinhada à proposta deste estudo, ao corresponsabilizar a comunidade para aprimorar a resolubilidade de agravos de saúde, como é o caso da violência direcionada à população transvestigênere e LGBTQIAPN+ (lésbicas, *gays*, bissexuais, transexuais, *queer*/questionando, intersexos, assexuais/arromânticas/agênero, pansexuais/polissexuais, não binárias e mais). A lógica da corresponsabilização permite que profissionais de saúde, ao trabalharem com a comunidade, discutam as violências presentes nas relações familiares, nas escolas, nos centros comunitários e nas instituições religiosas. Os serviços de saúde podem fomentar a construção de novos discursos sobre a população transvestigênere, problematizando e afastando o caráter de estranheza e monstruosidade presente nas lógicas sociais binárias. A capacidade da APS de fornecer distribuição hormonal e acompanhamento no território também é crucial. Isso evita a necessidade de deslocamento para ambulatórios especializados localizados apenas em grandes centros e com filas extensas.

A integralidade do cuidado, aliada ao trabalho em rede, posiciona a APS como um aliado potencial na realização de um trabalho transversal com a população transvestigênere. Essa abordagem é essencial para conscientizar e enfrentar a violência que essas pessoas enfrentam na sociedade. Atuação mais ativa e direcionada à população transvestigênere no âmbito da APS é crucial para abandonar as lógicas dicotômicas biomédicas e permitir que as identidades transvestigêneres existam fora do campo da monstruosidade abjeta.

Entre as limitações desta pesquisa, destaca-se que ela foi conduzida exclusivamente por pessoas cisgênero. Isso dificultou o acesso ao campo, dado o contexto de vulnerabilidade em que as pessoas transvestigêneres se encontram devido à transfobia socialmente imposta por pessoas cisgênero. Essa dinâmica pode ter sido reforçada pelas lógicas de poder que atravessam tanto as classes sociais quanto o contexto entre a pesquisadora e as participantes. Isso limitou um maior aprofundamento das questões, uma vez que o estudo utilizou entrevistas e grupos focais online como ferramentas de obtenção de dados. Embora o formato online tenha sido interessante por permitir que a pesquisa alcançasse mais pessoas do que seria possível presencialmente, ele também impediu um aprofundamento maior das questões tratadas. Isso se deu, em parte, pela dificuldade em observar sinais de conforto e desconforto durante as interações, o que levou a evitar exposição a possíveis violências. Como resultado, alguns aprofundamentos não foram realizados.

Em conclusão, violência contra pessoas transvestigêneres deve ser entendida não apenas em seu aspecto intencional, mas também nas formas não intencionais. Para enfrentar essa realidade, é fundamental que o princípio da integralidade seja efetivamente implementado nos serviços de saúde, com especial atenção a atenção primária.
